# Mechanical Fault Diagnosis Method of Disconnector Based on Parallel Dual-Channel Model of Feature Fusion

**DOI:** 10.3390/s25226933

**Published:** 2025-11-13

**Authors:** Chi Zhang, Hongzhong Ma, Tianyu Hu

**Affiliations:** School of Electrical and Power Engineering, Hohai University, Nanjing 211100, China; 230206030008@hhu.edu.cn (C.Z.); 220206030001@hhu.edu.cn (T.H.)

**Keywords:** disconnector, black-winged kite algorithm-variational mode decomposition-gated recurrent unit (BKA-VMD-GRU), recurrence plot-vision Transformer (RP-ViT), feature fusion, vibration signal, mechanical fault diagnosis

## Abstract

Mechanical fault samples of disconnectors are scarce, the fault types vary, and the self-evidence is weak, which leads to a lack of perfect fault diagnosis methods, and hidden defects cannot be found in time. To solve this problem, a mechanical fault diagnosis method for disconnectors based on a parallel dual-channel feature fusion model is proposed. Firstly, the optimal parameters for variational mode decomposition (VMD) are obtained using the black-winged kite algorithm (BKA). After the signal decomposition, the kurtosis values of each intrinsic mode function (IMF) are calculated, screened, and reconstructed. The reconstructed signal is input into the gated recurrent unit (GRU) to capture its time-series characteristics. Then, the vibration signal is generated by the recurrence plot (RP) to generate the atlas set and input into the vision Transformer (ViT) to extract its spatial characteristics. Finally, the time-series and spatial characteristics are fused, the multi-head self-attention mechanism is used for training, and softmax is used for fault classification. The measured data results show that the diagnostic accuracy of the model for mechanical fault types reaches 97.9%, which is 3.2%, 4.3%, 1.0%, 2.4%, 2.9%, 1.8%, 2.1%, 9%, and 7.5% higher than the other nine models numbered #2–#10, respectively, verifying its effectiveness and adaptability.

## 1. Introduction

Disconnectors are indispensable switching devices for power systems. They are generally installed at both ends of the circuit breaker, and their usage is two to three times that of the circuit breaker. As the power grid expands in size, the number of disconnectors also increases, and their safe and stable operation is crucial for the reliability of the power system [1,2]. Disconnectors operate primarily in outdoor environments and are eroded by harsh environmental factors such as wind, frost, rain, and snow for a long time, which has a significant impact on the mechanical characteristics of disconnectors. Taking 30,220 kV substations in a city as an example, from 2020 to 2024, 160 faults occurred in the disconnector and 85 mechanical faults occurred as the main fault type, accounting for 53.125%. This study focuses on the GW4 disconnector (The equipment is produced by Shandong Taikai Disconnector Co., Ltd., and put into use in Nanjing, China), which has the highest failure rate. The common mechanical fault types of GW4 disconnectors mainly include mechanism jam, mechanism looseness, and three-phase asynchrony [3], which are explained as follows: ash or rust can easily build up in the transmission mechanism, causing mechanism jam. The level of congestion progressively increases if it is not cleared in a timely manner, and the mechanism is broken in severe cases. After the disconnector is switched on and off several times, the bolts of the transmission mechanism become loose, which leads to mechanism looseness, resulting in abnormal vibration of the disconnector. During the installation and debugging of the disconnector, the vibration caused by improper operation leads to an abnormal position of the adjustable connecting rod, and three-phase asynchrony occurs, which damages the connecting rod [4,5].

The existing mechanical fault diagnosis methods are mainly divided into two categories, one is based on signal processing technology and combined with a classification algorithm for fault diagnosis, and the other is based on an artificial intelligence deep learning method for research. In the first type of method, vibration signal is the mainstream characteristic signal of mechanical fault diagnosis; compared with torque and motor current signals, it can capture the abnormal characteristics of mechanical faults more clearly [6]. Therefore, signal processing algorithms such as empirical mode decomposition (EMD) and VMD are widely used. Sun et al. [7] used the optimization algorithm to select the best parameters of VMD to achieve the purpose of adaptive signal decomposition, so as to solve the problem of modal aliasing caused by EMD. After signal processing, feature selection is carried out manually and then combined with the k-nearest neighbor (KNN), support vector machine (SVM), and other classification algorithms to realize the classification of faults [8,9]. The first type of method has resulted in significant breakthroughs in fault diagnostics. Nevertheless, in the actual operation of disconnectors, early hidden dangers are frequently not evident, and the fault features associated with various types of mechanical failures may be similar. The aforementioned method can distinguish relatively obvious fault types; however, it is useless to distinguish similar features between multiple fault types, which significantly affects the precision of fault identification.

To improve the fault recognition rate under complex fault conditions, the application of the second type of method has gradually become widespread. Deep learning algorithms combine signal processing, feature extraction, and fault diagnosis into a single model, thereby avoiding the shortcomings of the traditional methods [10]. In the field of mechanical fault diagnosis, deep learning algorithms are divided into two categories. The first category involves the transformation of one-dimensional time-series signals into two-dimensional images through methods such as RP, Gramian angular field (GAF), Markov transition field (MTF), and other methods, and then combine them with ViT, convolutional neural network (CNN), and other mainstream two-dimensional image processing models for feature extraction to achieve fault diagnosis. Zhang et al. [11] used continuous wavelet transform to convert one-dimensional signals into two-dimensional images, and then uses CNN-Transformer model to comprehensively capture multi-scale fault features, and used multi label classification to output corresponding fault labels. The second category involves using the temporal feature memory ability of the CNN, GRU, recurrent neural network (RNN), long short-term memory (LSTM), and other models to mine the spatial and temporal feature connections of one-dimensional time-series signals and realize fault classification [12]. Liao et al. [13] proposed a fault diagnosis method for a hydro generating unit based on one-dimensional CNN-GRU. Based on the data structure of time series, they can adaptively learn the data under different operating conditions through one-dimensional CNN-GRU, so as to further determine the fault category.

Deep learning has many methods in two types of applications of mechanical fault diagnosis. How to select the most suitable method becomes very important. In the first type of application, in the method of converting signal into image, the RP can extract effective features from complex nonlinear vibration signals and identify properties that cannot be obtained using conventional systems. It is also applicable when the signal sampling is small, and has more advantages in processing one-dimensional vibration signals than GAF and MTF [14,15,16]. In the image space feature extraction method, with the development of computer vision, a new deep learning architecture represented by ViT came into being. ViT is a deep learning model based on Transformer architecture that does not depend on the fixed order of the sequence. It can be operated in parallel and does not require consideration of the problem of gradient disappearance. Therefore, it has faster training speed and better performance [17]. It has more advantages than traditional deep learning models, such as CNN, in dealing with global information, flexibility of the model architecture, and efficiency of parallel computing. In the second type of application, the feature extraction methods for one-dimensional time-series signals are mainly based on the CNN, RNN, and variants of RNN, GRU, and LSTM. When capturing the sequence dependence between features, the CNN lacks the ability to model the relationship between targets, and the processing of all parts is equal and lacks pertinence. Although RNNs can process sequence data, they cannot be calculated in parallel and are unsuitable for training large-scale data sets [18]. As a variant of the RNN, LSTM inherits most of its characteristics and can address issues related to RNN convergence difficulty and gradient disappearance to some degree. Nonetheless, the structure of the LSTM is relatively complex and contains multiple gating mechanisms. It is difficult to save information at long intervals during the calculation process. The GRU is a variant of LSTM, and its expression ability in the network is almost as good as that of LSTM. However, compared with LSTM, the GRU improves the performance of the algorithm by simplifying the structure and reducing the parameters [19,20].

In order to better illustrate the contribution of this study, Table 1 briefly summarizes representative existing mechanical fault diagnosis methods for disconnectors, including their main advantages and limitations.

Considering the aforementioned issues, this study combines a one-dimensional time-series signal feature extraction model with a two-dimensional image space feature extraction model and proposes a disconnector mechanical fault diagnosis method based on a feature fusion parallel dual-channel model. This method uses BKA to obtain an optimal solution for the VMD parameters [*K*, *α*]. After the vibration signal is decomposed, the kurtosis values of each IMF are calculated, screened, and reconstructed, and the reconstructed signal is input into the GRU to capture its timing characteristics. Simultaneously, the vibration signal is generated by the RP and input into the ViT to extract its spatial features. Finally, the temporal and spatial features are integrated and trained by the multi-head self-attention mechanism, resulting in improved classification performance for the model. To confirm the efficacy of the proposed approach, its effects are verified through field experiments. At the same time, other related methods are added as experimental controls. The findings indicate that the proposed approach has better robustness and greater capacity for generalization. The diagnostic accuracy of the mechanical fault of the disconnector is higher than that of other methods.

## 2. Time-Series Feature Extraction Method Based on BKA-VMD-GRU

In this section, the time-series feature extraction method of disconnector vibration signals is discussed. Firstly, BKA is applied to determine the optimal VMD parameters for adaptive signal decomposition, and the decomposed IMF is screened and reconstructed based on kurtosis values to preserve the key components of the fault. Then, the reconstructed signal is input into the GRU to generate temporal features, effectively capturing the nonlinear and dynamic characteristics of the fault vibration signal. The detailed processes of BKA-VMD and GRU modeling are introduced in Section 2.1 and Section 2.2, respectively.

### 2.1. BKA-VMD

#### 2.1.1. VMD

VMD is an adaptive signal processing technique proposed in 2014, which achieves adaptive decomposition of signals by establishing a variational model and determining the optimal solution of constrained variations. The core idea is to decompose the original signal into several IMFs with limited bandwidth [21]. The variational problem is expressed as follows:

(1) $$\left\{\begin{array}{l} \min\left\{\sum_{k}{\left\|\partial_{t}\left[\left(\delta (t)+\frac{j}{\pi t}\right)\cdot u_{k}(t)\right]e^{-j\omega_{k}t}\right\|}_{2}^{2}\right\}\\ s.t.\sum_{k=1}^{K}f(t) \end{array}\right.$$

where *∂_t_* denotes the time-dependent partial derivative, *δ*(*t*) denotes the impulse function, · denotes the convolution operation, *u_k_*(*t*) denotes the decomposed IMF, *ω_k_* denotes the central frequency of the *k*-th mode, *K* denotes the number of IMF, *f*(*t*) denotes the initial signal. The objective of (1) is to minimize the total bandwidth of all IMFs while satisfying the signal reconstruction constraint. By performing Hilbert transform on each mode and moving it to the vicinity of the corresponding center frequency *ω_k_*, the model can effectively isolate different frequency components, thereby reducing mode aliasing.

For ease of solution, (1) is transformed into an unconstrained optimization form of (2) by introducing the quadratic penalty term *α* and Lagrange multiplier *λ*.

(2) $$L\left(\left\{u_{k}\right\},\left\{\omega_{k}\right\},\lambda \right)=\alpha \sum_{k=1}^{K}{\left\|\partial_{t}\left[\left(\delta (t)+\frac{j}{\pi t}\right)u_{k}(t)\right]e^{-j\omega_{k}t}\right\|}_{2}^{2}+{\left\|f(t)-\sum_{k=1}^{K}u_{k}(t)\right\|}_{2}^{2}+\left[\lambda (t),f(t)-\sum_{k=1}^{K}u_{k}(t)\right]$$

where (2) enables the problem to be solved using the Alternating Direction Multiplier Method (ADMM). The α is used to control the degree of bandwidth compression and improve decomposition stability. This theoretical mechanism makes VMD have a more rigorous mathematical foundation and better noise resistance compared to EMD. In the iterative solving process, each IMF solution is represented by *u_k_*(*t*), and it can be considered a minimization problem; the updated expression of the kth IMF in the frequency domain is as follows:

(3) $${\hat{u}}_{k}^{n+1}(\omega )\;=\;\frac{\hat{f}(\omega )-\sum_{i\neq k}{\hat{u}}_{k}(\omega )\;+\;\hat{\lambda }(\omega )/2}{1\;+\;2\alpha \left(\omega -\omega_{k}\right)}$$

where (3) represents that the *k*-th mode is determined by the residual of the current frequency band and is constrained by the center frequency to maintain narrowband characteristics, which facilitates the extraction of mechanical fault characteristic frequencies. The center frequency *ω_k_* of each IMF is the solution to the minimization problem. The formula for updating the *ω_k_* is as follows:

(4) $$\omega_{k}^{n+1}\;=\;\frac{\int_{0}^{\alpha }\omega {\left|{\hat{u}}_{k}(\omega )\right|}^{2}d\omega }{\int_{0}^{\alpha }{\left|{\hat{u}}_{k}(\omega )\right|}^{2}d\omega }$$

where (4) uses the weighted average energy method to locate the center frequency. The higher the energy, the greater the contribution of frequency components to the mode, giving the decomposition results clear and interpretable frequency meanings. The update of the Lagrange multiplier value is expressed as follows:

(5) $${\hat{\lambda }}^{n+1}(\omega )\;=\;{\hat{\lambda }}^{n}(\omega )\;+\;\tau \left[\hat{f}(\omega )\;-\sum_{k}{\hat{u}}_{k}^{n+1}(\omega )\right]$$

where (5) ensures that the sum of each IMF gradually approaches the original signal, suppressing the accumulation of errors during the decomposition process. When the iterative result satisfies (6), the operation process is completed, and the value of *K* is obtained after the Fourier transform.

(6) $$\sum_{k}\frac{{\left\|{\hat{u}}_{k}^{n+1}-{\hat{u}}_{k}^{n}\right\|}_{2}^{2}}{{\left\|{\hat{u}}_{k}^{n}\right\|}_{2}^{2}}\;<\;\varepsilon ,\;\varepsilon \;>\;0$$

where (6) indicates that when the modal changes between adjacent iterations are minimal, VMD obtains a stable solution. The final multiple IMF components will be used for subsequent kurtosis value calculation and signal reconstruction, providing high-quality input for GRU feature extraction.

#### 2.1.2. BKA

Inspired by the predatory mobility of black-wing kites and the wisdom of group migration, BKA was proposed in 2024 and its process is summarized as follows:

The process of population initialization involves ensuring that each black-winged kite is distributed uniformly in its initial position [22].

(7) $$X_{i}=\mathrm{lb}+\mathrm{rand}\left(\mathrm{ub}-\mathrm{lb}\right)$$

where *X_i_* denotes the location of the *i*-th black-winged kite, *i* denotes an integer within the range of 1 to *N*, *N* denotes the population, rand denotes a random number between 0 and 1, and *ub* and *lb* denote the upper and lower limits of the search range, respectively.

The attack behavior of the black-winged iris is used for the global search, which is expressed as follows:

(8) $$x_{t+1}^{i,j}=\left\{\begin{array}{ll} x_{t}^{i,j}\;+\;n\;\times \;(1\;+\sin(r))\;\times \;x_{t}^{i,j} & p\;<\;r\\ x_{t}^{i,j}\;+\;n\;\times \;(2r-1)\;\times {\;x}_{t}^{i,j} & \mathrm{else} \end{array}\right.$$

where *n* = 0.05 × exp[−2 × (*t*/*T*)^2^], *t* is the current iteration count, *T* is the maximum iteration count, $x_{t}^{i,j}$ represents the *j*-th dimension position of the *i*-th black-winged kite at the *t*-th time, *p* is 0.9, and *r* denotes a random number between 0 and 1.

The migration behavior of black-winged kite is expressed as follows:

(9) $$x_{t+1}^{i,j}=\left\{\begin{array}{ll} x_{t}^{i,j}\;+\;C(0,1)\;\times \;\left(x_{t}^{i,j}-L_{t}^{j}\right) & F_{i}\;<{\;F}_{\mathrm{ri}}\\ x_{t}^{i,j}\;+\;C(0,1)\;\times \;\left(L_{t}^{j}-m\;\times \;x_{t}^{i,j}\right) & \;\;\mathrm{else} \end{array}\right.$$

where *m* = 2 × sin(*r* + π/2), $L_{t}^{j}$ represents the leading scorer (the current optimal solution) of the *j*-dimensional black-winged kite in the *t*-th iteration, *F_i_* is the fitness value of the current individual, *F_ri_* represents the fitness value of the *j*-dimensional random position obtained by any black-winged kite in the *t*-th iteration, and C(0,1) represents the Cauchy variation centered at the origin.

**Figure 1:**
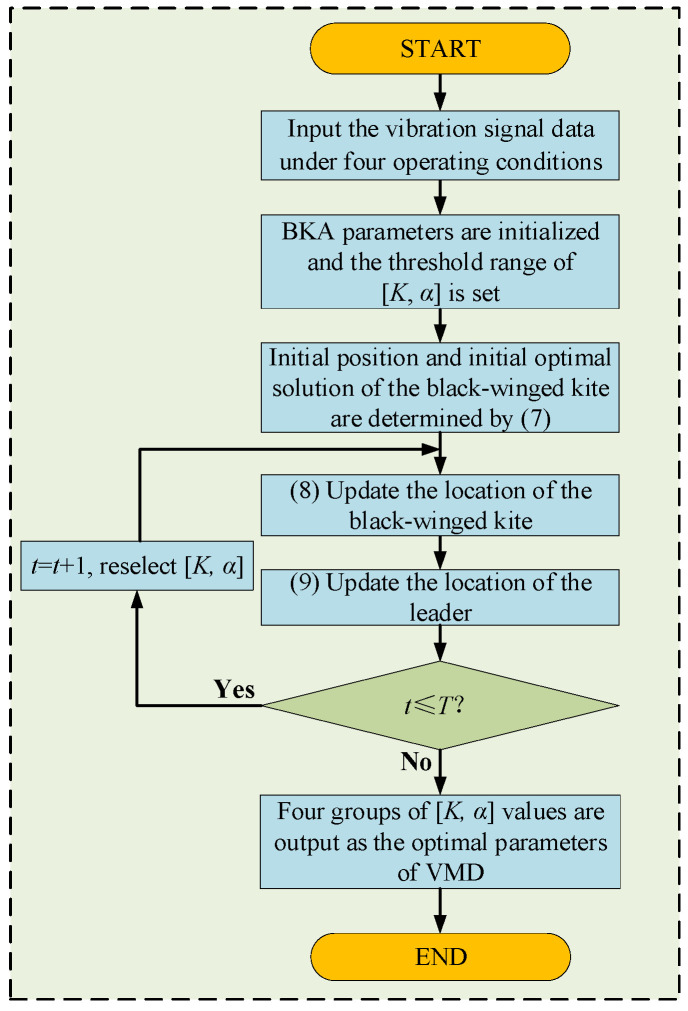
Process diagram of BKA-VMD.

In this study, the BKA is used to select the optimal *K* and *α* values for the VMD. The steps of the algorithm are as follows, the specific flowchart is shown in Figure 1.

1. Initialize population parameters: Including population number *N* and iteration number *I_ter_*, etc.
2. Fitness function: The envelope entropy serves as the fitness function for optimizing the VMD parameters, with the fitness value of each individual and optimal position of the initial population calculated accordingly.
3. Attack behavior: The new solution is explored by randomly disturbing the current solution, and the location of the black-winged kite is updated.
4. Migration behavior: Evaluate the fitness value of each black-winged kite, dynamically select the leader, adjust the search direction, and use random changes generated by the Cauchy distribution to update the location.
5. Update the population: Update all black-winged kite positions in the population according to the leader position to ensure that the population is concentrated in a better solution domain.
6. Steps (2)–(5) are repeated until the termination condition is satisfied.
7. The optimal fitness and individual position are calculated, and the optimal *K* and *α* are the outputs.

### 2.2. GRU

**Figure 2:**
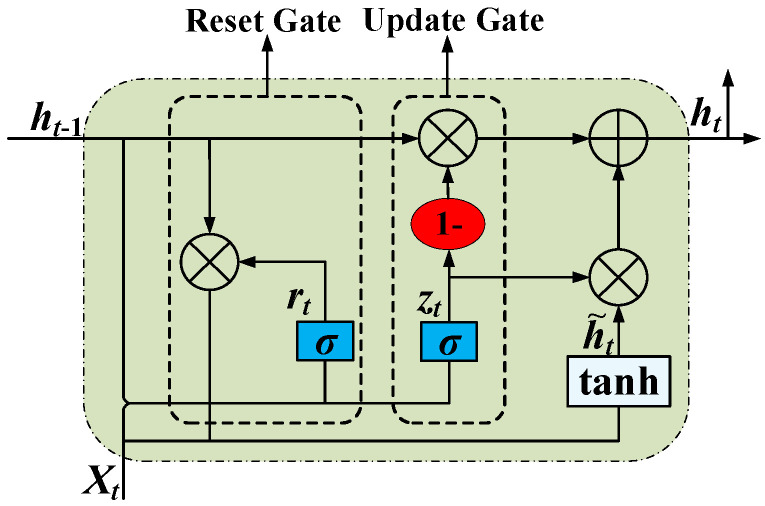
Basic structure of GRU.

GRU is an optimization of LSTM that controls the retention of information by merging the input gate and forgetting gate in LSTM into an update gate and introducing a reset gate to determine the combination of old and new information, thereby improving the efficiency of sequence data processing [23,24]. Its structure is shown in Figure 2.

GRU is mainly composed of the following parts:

1. Two inputs, *h_t_*_−1_ and *x_t_*, represent the output of the neuron at the previous moment and the output of the neuron in the previous layer, respectively.
2. Two doors, namely, the update door *z_t_* and the reset door *r_t_*. The update gate and reset gate are activated by function *σ* at time *t*, which is calculated as follows:

(10) $$\left\{\begin{array}{c} z_{t}=\sigma (W^{z}x_{\;t}+{\;U}^{z}h_{t-1})\\ r_{t}=\sigma (W^{r}x_{t\;}+U^{r}h_{t-1}) \end{array}\right.$$

where *σ* is the sigmoid activation function, *W*^z^ and *U*^z^ are the weights of the update gate, and *W*^r^ and *U*^r^ are the weights of the reset door.

3. A state, that is, the internal state of the neuron at time *t*, can be expressed as follows:

(11) $${\tilde{h}}_{t}=\tanh(r⊙Uh_{t-1}+Wx_{t})$$

where tanh is a hyperbolic tangent activation function, *U* and *W* are the weights of ${\tilde{h}}_{t}$, *r* is the state of reset door, and ⊙ denotes the Hadamard product.

4. An output, which is the final output, can be expressed as follows:

(12) $$h_{t}=(1-z_{t})⊙{\tilde{h}}_{t}+z_{t}⊙h_{t-1}$$

The vibration signal has complicated frequency components and pronounced nonlinear features, and the direct input into the GRU for time-series signal feature extraction cannot fully reflect the characteristics of the different mechanical fault types. Therefore, the best [*K*, *α*] is selected in the BKA-VMD algorithm, after which the kurtosis value is introduced into the obtained IMF component for evaluation, and the IMF component containing important information is selected for signal reconstruction to obtain a one-dimensional time-series characteristic signal. The reconstruction process is briefly described as follows:

1. Determine the kurtosis value for each IMF.

(13) $${\mathrm{Kurtosis}}_{i}=\frac{E[(IMF_{i}-\mu_{i}{)}^{4}]}{{\sigma_{i}}^{4}}$$

where *σ_i_* and *μ_i_* are the standard deviation and mean vector of the IMF, respectively, and *i* = 1, ···, *K*, *E* are the expected values of the signal.

2. When the kurtosis value exceeds three, the IMF is a fault signal containing more impact components. Therefore, IMFs with kurtosis values greater than three are selected for reconstruction, and the selected IMFs set is as follows:

(14) $$\Omega =\left\{IMF_{i}|{\mathrm{Kurtosis}}_{i}\;>\;3\right\}$$

3. The reconstructed signal is obtained as follows:

(15) $$\tilde{x}=\sum_{{\mathrm{IMF}}_{i}\in \Omega }{\mathrm{IMF}}_{i}$$

**Figure 3:**
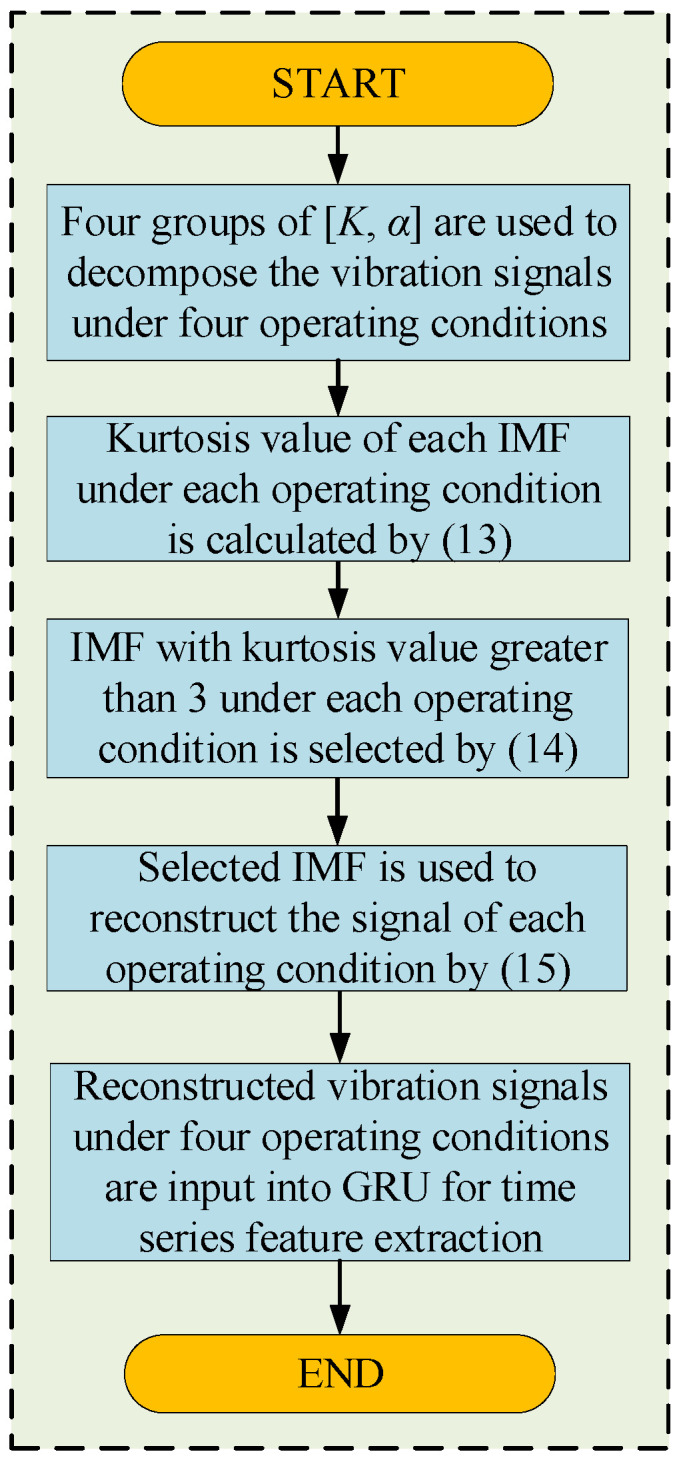
Method flow of time-series feature extraction.

If the kurtosis value of the IMF obtained through decomposition is less than three, the IMF that exhibits the highest kurtosis value is chosen as the reconstructed signal. Finally, the reconstructed vibration signal is input into the GRU for time-series feature extraction. Figure 3 shows a specific procedure for extracting time-series features.

## 3. Spatial Feature Extraction Method Based on RP-ViT

### 3.1. RP

In nonlinear dynamical systems, the evolution of vibration signals can be seen as reconstructing trajectories in phase space. When a device enters a fault state, its motion usually exhibits periodic, stable, and non-stationary transition behavior changes, and RP provides a method for visualizing these dynamic characteristics, which can extract spatial features that are difficult to observe in the original one-dimensional signal.

Compared with traditional time–frequency analysis, RP is more sensitive to weak and transient nonlinear fault characteristics. It not only preserves the amplitude and phase information of the original one-dimensional signal, but also enhances the display of fault characteristics, making fault information more abundant. Therefore, converting vibration signals into two-dimensional RP images can effectively enrich information representation and provide more discriminative inputs for the spatial feature extraction of ViT models [25]. The precise steps are as follows:

1. The phase space of the time series *x_i_* (*i* = 1, 2, ···, *N*) is reconstructed, *m* and *τ* are determined by the error nearest neighbor method and autocorrelation function method, respectively, and each phase point *X_i_* in the phase space is calculated as follows:

(16) $$X_{i}=[x_{i},x_{i+\tau },\cdots ,x_{i+(m-1)\tau }]$$

2. The distance *L_i_*_,*j*_ between the phase points *X_i_* and *X_j_* is obtained as follows:

(17) $$L_{i,j}=∥X_{i}-X_{j}∥,\;i,j=1,\cdots ,N$$

3. According to the difference between the selected threshold *ε* and *L_ij_*, the result is input into the Heaviside function to obtain the value of each point in the recursive matrix.

(18) $$R_{\mathrm{ij}}=\mathrm{Heaviside}(\varepsilon -L_{\mathrm{ij}}),\;i,j=1,\cdots ,N$$

Here the Heaviside function is expressed as follows:

(19) $$\mathrm{Heaviside}(x)\;=\;\left\{\begin{array}{c} 1,x\;⩾\;0\\ 0,x\;<\;0 \end{array}\right.$$

4. For each recursive point that satisfies the condition, a black point is marked in the coordinate system and a white point is marked for *R_ij_* = 0. Because *R_ij_* = *R_ji_* and *R_ij_* = 1 (*i* = *j*), the characteristic of RP is that there is an obvious main diagonal, and the entire graph is symmetrical about this main diagonal.

### 3.2. ViT

**Figure 4:**
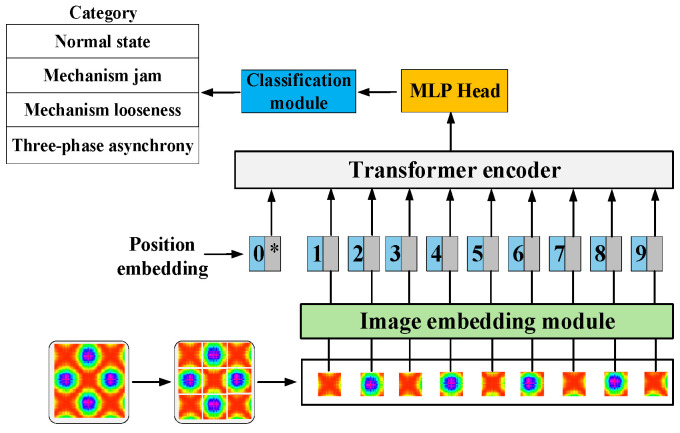
Overall structure of ViT.

ViT is a model that utilizes Transformer architecture specifically for image classification. It realizes image recognition by image segmentation and embedding, and then uses a Transformer encoder to process this information [26]. The overall structure is shown in Figure 4.

#### 3.2.1. Image Embedding Module

Suppose that the input image *x* ∈ R*^HWC^*, it is converted into a two-dimensional image block sequence with dimensions *N* × (*P*^2^ × *C*), where the original image dimensions are (*H* × *W*), and the number of channels is *C*. The dimensions of the image block generated by this method is (*P*, *P*), and the total number of blocks is *N* = *HW*/*P*^2^. The number of created blocks corresponds to the effective input sequence length of the ViT network. In this layer, a hidden vector of size *D* is used to flatten the image blocks, and trainable linear mapping is used to map *P*^2^ × *C* to *D* dimensions, while keeping the number of image blocks *N* unchanged [27]:

(20) $$z_{0}\;=\;[x_{cls};x_{P}^{1}E;x_{P}^{2}E;\cdots ;x_{P}^{N}E]\;+\;E_{\mathrm{pos}}$$

where *E* is the matrix used to realize the linear mapping; *E_pos_* is position embedding; $x_{\mathrm{cls}}$ represents 0* in Figure 4, because when the input picture is cut into multiple patches, each patch will be converted into a vector through linear mapping, that is, the blue block 1~9 in Figure 20. In addition to these patches, the model will also add a special token at the front, called CLS token, that is $x_{\mathrm{cls}}$.

#### 3.2.2. Transformer Encoder

**Figure 5:**
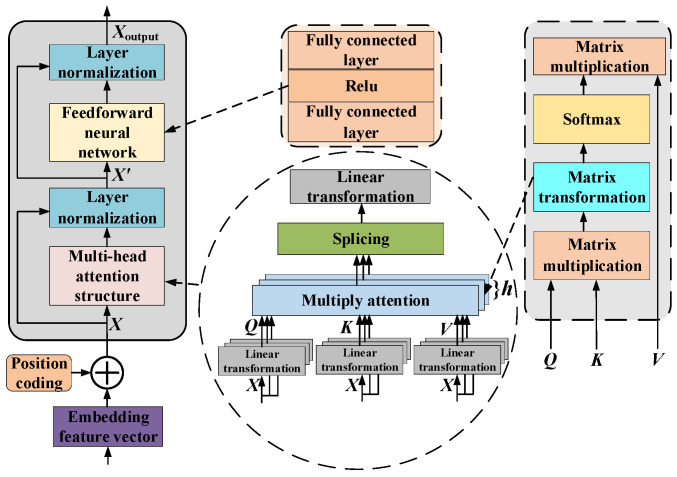
Basic structure of Transformer encoder block.

The Transformer encoder is stacked with multiple identical modules, and Figure 5 illustrates its internal structure, which mainly includes a multi-head self-attention layer and a feed-forward neural network layer. To enhance the model’s accuracy and augment the network depth, each sub-layer is interconnected by residual and layer normalization [28].

1. Multi-head self-attention operation layer

The self-attention operation module serves as the fundamental component of the Transformer encoder. As shown in Figure 5, through parallel *h*-attention calculations, multiple parameter-independent representation subspaces are obtained through parallel *h*-attention calculations. Input matrix *X* ∈ $R^{s\times d_{\mathrm{model}}}$ is mapped to query matrix *Q*, key matrix *K*, and value matrix *V* using three linear parameter transformation matrices, where *s*: is the number of patch embeddings and *d*_model_ is the feature dimension of each patch:

(21) $$\left\{\begin{array}{c} Q\;=\;W^{Q}X\\ K\;=\;W^{K}X\\ V\;=\;W^{V}X \end{array}\right.$$

where *W^Q^*, *W^K^*, *W^V^* ∈$R^{d_{\mathrm{model}}\times d_{k}}$; *Q*, *K*, *V* ∈ $R^{s\times d_{k}}$; *W^Q^*, *W^K^*, *W^V^* map *X* from the *d*_model_-dimension to the *d_k_*-dimension space, and *d_k_* is the dimension of the key matrix.

The formula for calculating multi-head self-attention is as follows:

(22) $$\left\{\begin{array}{l} A_{\mathrm{ttention}}(Q,K,V)\;=\;F_{\mathrm{softmax}}(\frac{QK^{T}}{\sqrt{d_{k}}})V\\ h_{\mathrm{ead}i\;}={\;A}_{\mathrm{ttention}}(Q_{i},K_{i},V_{i})\\ M_{\mathrm{ultiHead}}(Q,K,V)\;=\;C_{\mathrm{oncat}}(h_{\mathrm{ead}1},h_{\mathrm{ead}2},\cdots ,h_{\mathrm{ead}i})W^{O} \end{array}\right.$$

where *i* represents the dimension subscripts of the feature vector. The point multiplication attention mechanism is a feature fusion operation. The softmax function *F*_softmax_(·) is beneficial for the back-propagation gradient calculation, and the result is smoothed to a 0–1 interval. The attention distribution in the *d_k_*-dimensional space of multiple groups is concatenated by *C*_oncat_(·), and the final attention-layer output is acquired using the weight matrix *W*^O^ transformation. The dimensions of several matrices are explained as follows: *Q_i_*, *K_i_*, *V_i_* ∈$R^{s\times d_{k}}$; *A*_ttention_ (*Q*, *K*, *V*) ∈$R^{s\times s}$; *h*_ead*i*_ ∈$R^{s\times d_{k}}$; C_oncat_(*h*_ead1_, *h*_ead1_,···, *h*_ead*i*_) ∈$R^{s\times (h\cdot d_{k})}$;$W^{O}$∈$R^{(h\cdot d_{k})\times d_{\mathrm{model}}}$; *M*_ultiHead_(*Q*, *K*, *V*) ∈$R^{s\times d_{\mathrm{model}}}$.

The attention weight formula is as follows:

(23) $$A_{\mathrm{ij}}\;=\;F_{\mathrm{softmax}}(Q_{i}K_{j}^{T}/\sqrt{d_{k}})$$

where *A_ij_* represents the attention weight between the different features. When extracting the attention distribution of a unit, all measurement information of the unit is integrated as a feature of the unit for the attention operation, and an attention distribution map is obtained. Therefore, the output of the data after the self-attention operation layer is regarded as the weighted sum of all the features in the feature map. The self-attention operation realizes direct point multiplication fusion between different position features, which is not affected by the distance between features, enhances the feature representation, and has a global receptive field, so that more information can be maintained under data pollution.

2. Feed-forward neural network layer

The feed-forward neural network layer is composed of two layers of fully connected networks, each of which is linearly mapped to the input vector, whereas the hidden layer in the middle is activated by the ReLu function. The formula for a feed-forward neural network is as follows:

(24) $$F_{\mathrm{FN}}(x)\;=\max\left(0,xW_{1}+b_{1}\right)W_{2}+b_{2}$$

where *x* represents the normalized output vector derived from the attention layer; *W* denotes the weight vector; *b* denotes the bias term; *W*_1_ ∈ $\;R^{d_{\mathrm{model}}\times d_{\mathrm{ff}}}$; *W*_2_∈$R^{d_{\mathrm{ff}}\times d_{\mathrm{model}}}$; and *F*_FN_(*x*) ∈${\;R}^{s\times d_{\mathrm{model}}}$.

The MLP Head is composed of a fully connected layer and a nonlinear activation function, which is responsible for processing the weighted output transmitted by the multi-head self-attention mechanism to identify and classify different fault types. Finally, the one-dimensional time-series vibration signal is converted into a two-dimensional recursive image and input into ViT for spatial feature extraction.

## 4. Parallel Dual-Channel Model Based on Feature Fusion

### 4.1. Construction of Model

**Figure 6:**
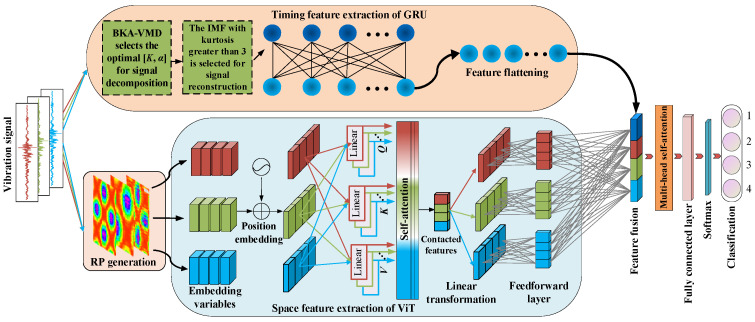
Parallel dual-channel model based on feature fusion.

To enhance the precision of fault diagnosis with a single timing signal input, this study constructs a timing-image fusion diagnosis model based on BKA-VMD-GRU and RP-VIT, as shown in Figure 6.

As typical time-series data, the vibration signal contains obvious periodicity and dynamic change law. GRU can effectively capture the time dependence, so as to extract the time-series characteristics sensitive to faults. At the same time, the vibration signal is transformed into a two-dimensional image by using the recursive graph method, and the global energy distribution and state evolution mode are mapped. ViT relies on the self-attention mechanism to mine cross-regional dependencies and extract more robust spatial features. Therefore, the temporal features and spatial features are complementary in information expression, and the fusion can more fully reflect the characteristics of vibration signals and improve the accuracy and robustness of fault diagnosis under complex working conditions.

The model shown in Figure 6 is roughly divided into three parts: the upper channel layer, the lower channel layer, and the recognition layer. The upper channel layer is the BKA-VMD-GRU model, and the lower channel layer is the RP-VIT model. The recognition layer includes a feature fusion layer, a multi-head self-attention layer, a fully connected layer, and a softmax classification layer. The specific operation process of the model is as follows:

Firstly, the optimal parameters [*K*, *α*] of the VMD are selected by BKA, and the IMFs are obtained by the VMD of the vibration signal. The kurtosis of each IMF is calculated and screened. The selected IMF is reconstructed to obtain a one-dimensional time-series feature signal, which is flattened and input into the GRU to extract dynamic time-series features. Secondly, the vibration signal is transformed into a color image by RP image coding, and the compact spatial features are obtained after patch embedding and Transformer coding. Then, according to the size of the array, the element-by-element addition method is used to stitch, fuse, and align the obtained spatio-temporal features at the addition layer. Thus, the image pattern can be recognized, the long-term dependence of the signal can be understood, and a comprehensive signal view can be provided. Finally, in order to improve the ability of extracting fault feature information, a multi-head self-attention mechanism is introduced to adaptively assign different weights to various features, focusing on strengthening features, further modeling the correlation between time and space features, realizing cross-modal interaction, and improving the accuracy of fault identification. The softmax layer is the input for classification, and the final result is the output.

### 4.2. Fault Diagnosis Process

**Figure 7:**
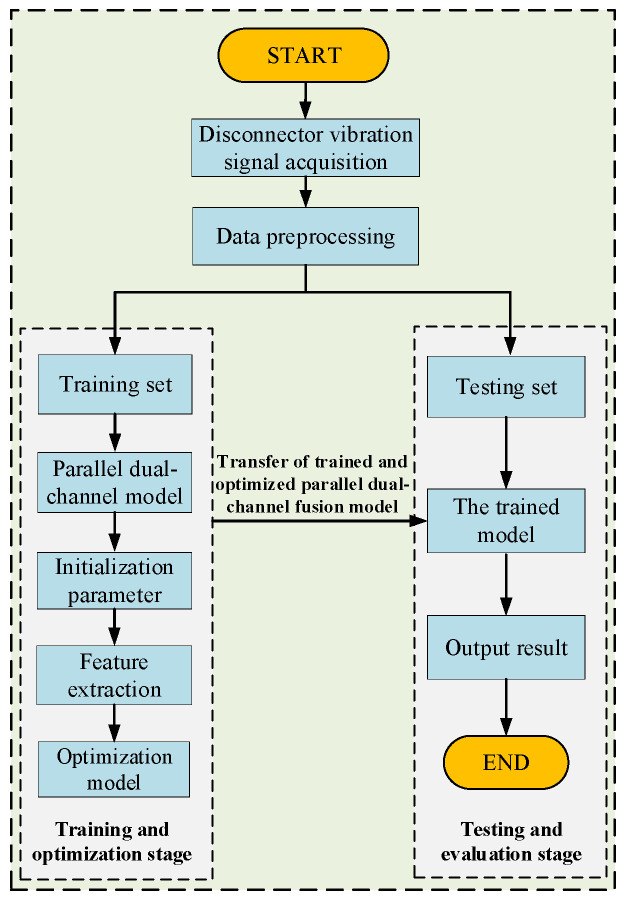
Flowchart of mechanical fault diagnosis for the disconnector.

The overall process of the parallel dual-channel model based on the feature fusion proposed in this study is shown in Figure 7.

1. Signal preprocessing and data set division: The collected original vibration signal is denoised. Considering the limited amount of data collected from the mechanical fault data set of the disconnector, the random differentiation of the training set and the test set may lead to an unbalanced sample distribution and an inability to obtain accurate training results. Therefore, the five-fold cross-validation method is selected to train the model. This method divides the data set into five mutually exclusive parts on average. Each iterative training takes four parts in turn as the training set, and the remaining one is used as the test set. After five repetitions, it is ensured that each data has played the role of training set and test set, so as to comprehensively evaluate the model, reduce the errors caused by different divisions, and improve the stability and reliability of evaluation. Finally, the mean of the five training results is obtained as the estimation of the final accuracy of the algorithm.
2. Model training: The training data set is entered into the constructed model for feature extraction, and iterative training is conducted by adjusting the model structure, learning rate, and other hyperparameters until the model satisfies the diagnostic accuracy requirements.
3. Model evaluation: The trained model receives the test data set as input, and the model evaluation index values are calculated to obtain the final diagnosis result.

## 5. Experiments and Result Analysis

### 5.1. Mechanical Fault Experiment of Disconnector

#### 5.1.1. Construction of Experimental Platform

**Figure 8:**
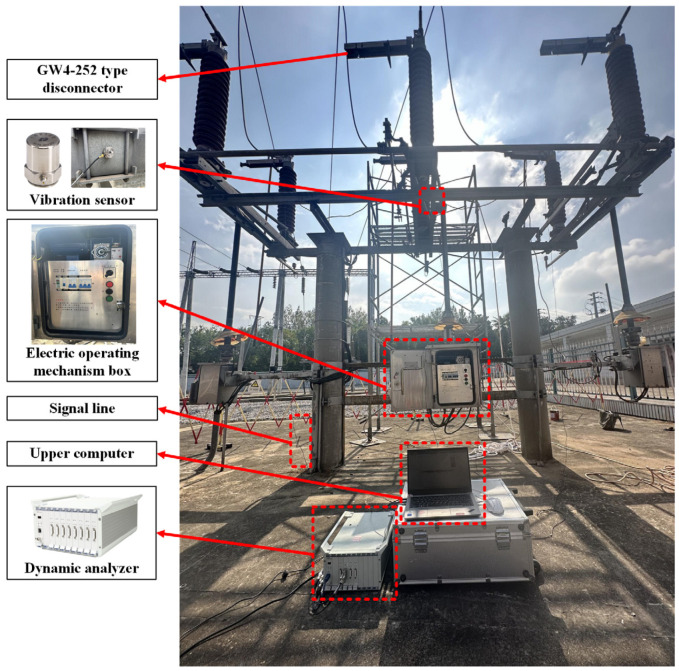
Vibration experimental platform of a GW4-252 type disconnector.

In this experiment, a GW4-252 disconnector (The equipment is produced by Shandong Taikai Disconnector Co., Ltd., and put into use in Nanjing, China) at a 220 kV substation is the subject of the test, and the vibration experimental platform of the disconnector is built, which mainly includes the disconnector equipment, dynamic analyzer, vibration sensor, electric operating mechanism box, upper computer, and signal lines, as shown in Figure 8.

Vibration sensors are typically categorized into displacement, speed, and acceleration sensors. Frequency range plays a significant role in the selection of these sensors. In this study, we focus on an acceleration sensor that is more sensitive to mechanical vibrations and covers a wider frequency range. To achieve this, the 1A212E piezoelectric acceleration sensor (The equipment is produced by Jiangsu DongHua Testing Technology Co., Ltd., and put into use in Nanjing, China) is selected, which is characterized by its small size, wide frequency response range, large measurement range, good linearity, and high sensitivity.

In this experiment, the DH5922D dynamic analyzer (The equipment is produced by Jiangsu DongHua Testing Technology Co., Ltd., and put into use in Nanjing, China) is selected. The system has a built-in 24 V/4 mA bias circuit, which can be adapted to the 1A212E piezoelectric acceleration sensor and collects its output signal to perform the test and analysis of the vibration signal. The device can record multi-channel signals continuously in real time, and each channel works synchronously in parallel during the acquisition period and has strong anti-interference ability and stability.

#### 5.1.2. Layout of Vibration Sensor and Faults Setting

**Figure 9:**
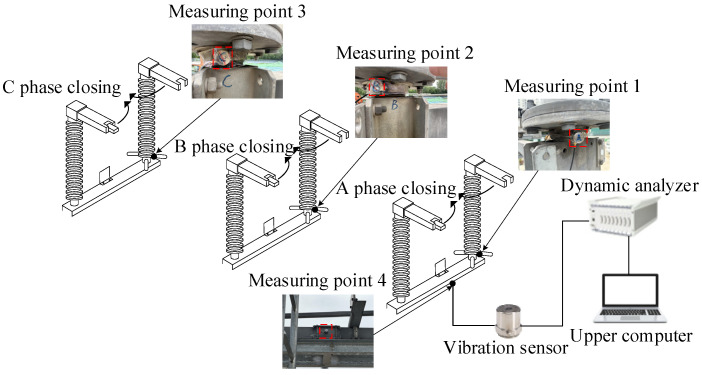
Installation position of vibration sensors.

The vibration sensor should be positioned close to the vibration source to ensure a clear reflection of the vibration events, minimize attenuation, reduce interference from the surrounding components, and enhance the reliability of the events indicated by the acquired vibration signal. The preset installation locations of the vibration sensors are illustrated in Figure 9, divided into measuring points 1–4, which are installed in the left pole bracket of the A, B, and C phases, and the middle of the beam of the A phase. Through experiments, it is found that under the fault method set in this study, the vibration signal intensity and information amount recorded by the vibration sensor at measuring point 4 are the largest. Finally, after communicating with the technicians, the vibration sensor is designated for installation at measuring point 4, which may indicate various mechanical faults and does not influence the operation of the disconnector opening and closing mechanism. The 1A212E vibration sensor (The equipment is produced by Jiangsu DongHua Testing Technology Co., Ltd., and put into use in Nanjing, China) selected for the test is adsorbed onto the beam of phase A through the magnetic base.

**Figure 10:**
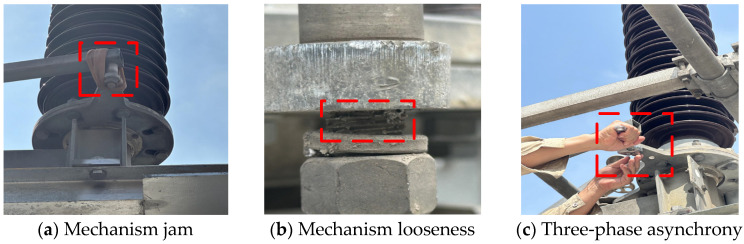
Fault simulation methods.

Figure 10 illustrates the methods used for fault simulation. The subgraphs (a), (b), and (c) simulate mechanism jam, mechanism looseness, and three-phase asynchrony, respectively, and all the simulation methods are performed without destroying the equipment itself. In Figure 10a, the method involves simulating jamming severity by attaching varying quantities of elastic rubber rings to the joint components of the transmission mechanism. In Figure 10b, the method involves loosening the A-phase from the moving pole arm joint bolt. In Figure 10c, the method involves adjusting the A phase from the polar arm to ensure it lags behind the B and C phases.

#### 5.1.3. Experimental Procedure

**Figure 11:**
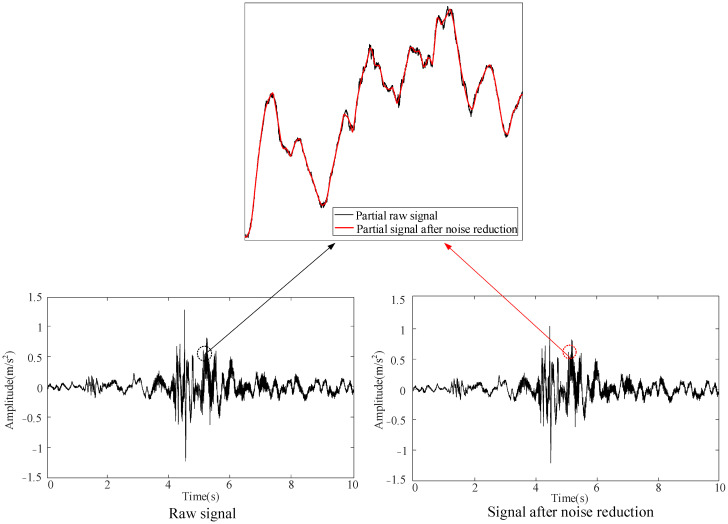
Signal noise reduction process under normal state.

The dynamic analyzer features a sample frequency of 5 kHz, and each operating condition is marked simultaneously: the normal state, mechanism jam, mechanism looseness, and three-phase asynchrony are labeled 1–4, respectively. Each operating condition tests 250 datasets for a total of 1000 datasets. The one-time closing is recorded as a set of data; the closing procedure takes approximately 10 s, and the collected data is automatically exported to an Excel file format. The collected data is preprocessed using the wavelet packet algorithm for noise reduction [29]. Figure 11 shows the signal noise reduction process in the normal state.

**Figure 12:**
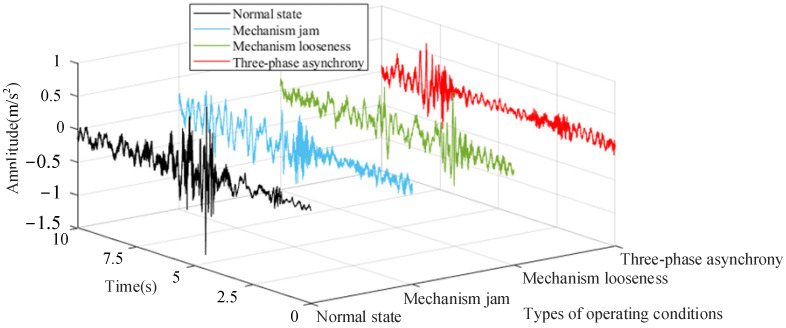
Vibration signal after noise reduction under the four operating conditions.

It can be seen from Figure 11 that the noise reduction effect is very obvious. The vibration signals following preprocessing under the four operating conditions are shown in Figure 12.

**Figure 13:**
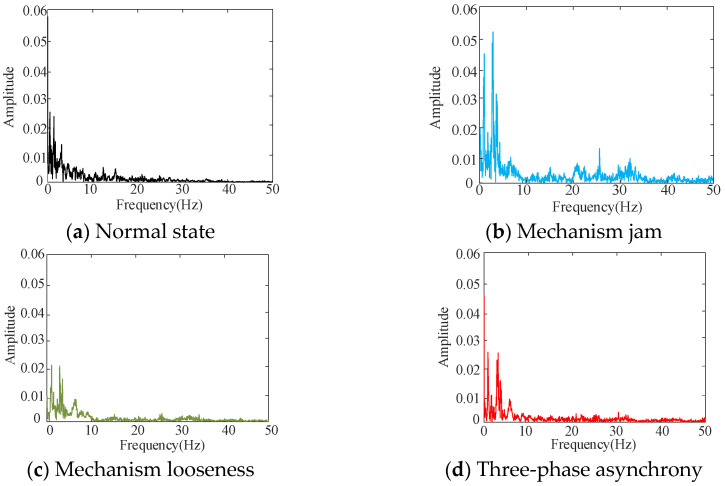
Spectrum of vibration signal.

Figure 12 illustrates that the vibration signals exhibit variations under different mechanical states within the time domain; however, it is difficult to discern the signal features from the time domain. Consequently, the primary frequency distribution is obtained using Fourier transform, and the results are presented in Figure 13.

**Figure 14:**
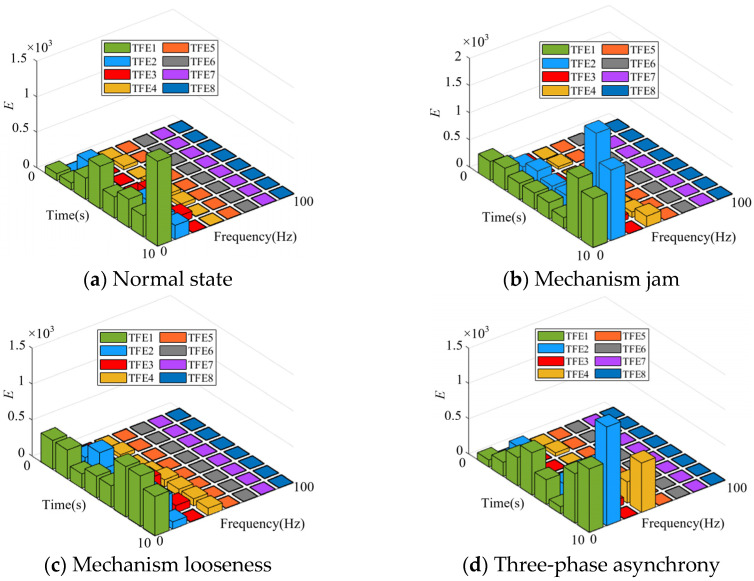
Time–frequency energy values of vibration signals.

Figure 13 illustrates that the primary frequency range of the vibration signal is within 50 Hz. To quantitatively reflect the changes in the vibration signals of different faults in time, frequency, and amplitude, the wavelet packet transform is used to calculate the time–frequency energy of vibration signals in four different states. The number of wavelet packet decomposition layers is three, and the sampling frequency is 200 Hz. According to Nyquist’s theorem, the signal frequency is less than 100 Hz. After decomposition, the frequency band component with an equal fraction of eight is obtained, and the frequency range is 0–100 Hz. The time–frequency energy of the vibration signal is calculated and the distribution (TFE1-TFE8) is illustrated in Figure 14.

The time–frequency energy is focused within 50 Hz under varied operating conditions, as shown in Figure 14. In Figure 14, from a numerical perspective, the energy value of mechanism jam is higher. The output power of the motor increases when a jam occurs, resulting in heightened equipment vibration and an increase in energy value. The energy value of mechanism looseness is lower than that under other operating conditions. This is because when the mechanism is loose, the gap increases, resulting in a decrease in the vibration frequency of the equipment and energy value. Under the other two conditions, the energy values are relatively close.

The above analysis indicates that the vibration waveforms exhibit distinct characteristics under various fault conditions. It is possible to differentiate between various fault states by extracting the timing and spatial features of vibration waveforms.

### 5.2. Result Analysis

#### 5.2.1. Analysis of Timing Feature Extraction

VMD is optimized using BKA, and the initial parameters are listed in Table 2.

**Figure 15:**
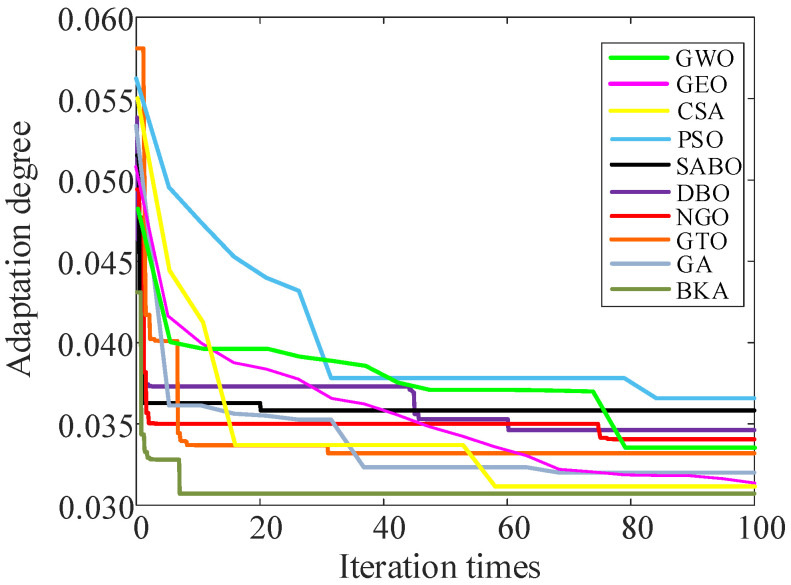
Fitness iteration curves of five algorithms.

BKA is compared with GWO (Grey Wolf Optimizer), GEO (Golden Eagle Optimizer), CSA (Crow Search Algorithm), PSO (Particle Swarm Optimization), SABO (Subtraction-Average-Based Optimizer), DBO (Dung Beetle Optimizer), NGO (Northern Goshawk Optimization), GTO (Gorilla Troops Optimizer), and GA (Genetic Algorithm) to verify its optimization performance. Figure 15 shows the fitness iteration curves for the five algorithms. The analysis indicates that as the number of iterations increases, the fitness curve of BKA demonstrates a trend of rapid convergence. It can quickly lock the globally optimal solution with fewer iterations, and has higher stability and better optimization efficiency.

After running the BKA-VMD algorithm, the *K* and *α* values of each data group at minimum envelope entropy are documented. The parameter optimization results under the four operating conditions are listed in Table 3.

**Figure 16:**
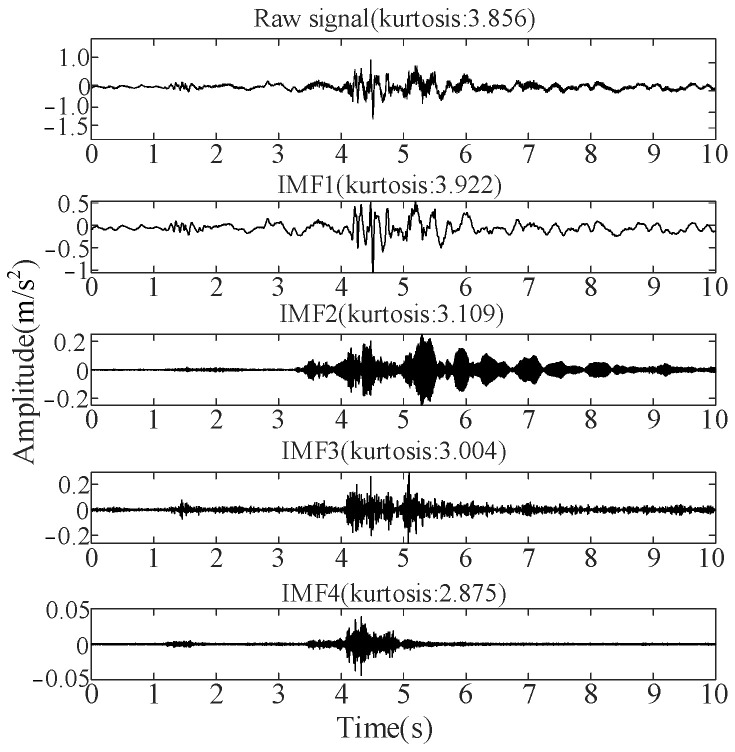
VMD results and kurtosis values corresponding to each IMF.

[*K*, *α*] in Table 3 is used as the initial parameter of VMD for signal decomposition. Using the vibration signal in the normal state as an example, the results of the VMD and kurtosis values corresponding to each IMF component are shown in Figure 16.

**Figure 17:**
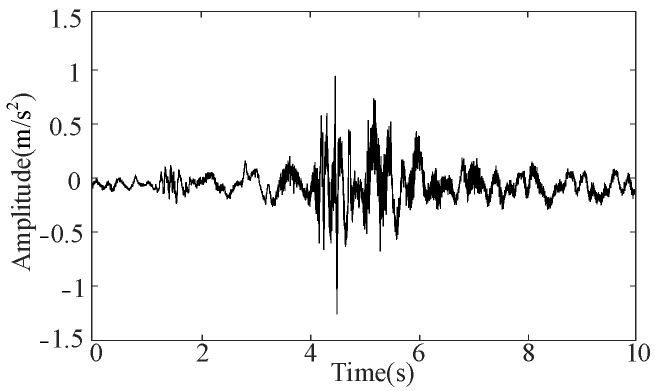
Reconstruction signals of IMF1~IMF3.

Figure 16 shows that the kurtosis values for IMF1, IMF2, and IMF3 exceed 3. The three selected IMFs are reconstructed under the normal state, and Figure 17 shows the reconstructed signal. Its kurtosis value is approximately 3.991, which is marginally higher than that of the original signal, indicating that it has stronger impact characteristics. Finally, the vibration signal samples collected under the four operating conditions are reconstructed and inputted into the GRU model for time-series feature extraction.

#### 5.2.2. Analysis of Spatial Feature Extraction

**Figure 18:**
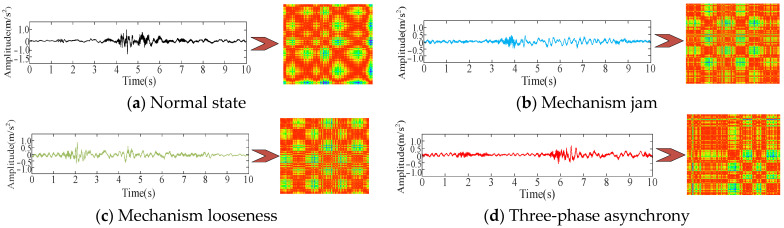
Partial two-dimensional recurrence plot.

The one-dimensional vibration time-series signals collected by the experiment under the four operating conditions are transformed into two-dimensional recurrence plots. A few of the plots are shown in Figure 18.

Before using ViT to extract the spatial features of the two-dimensional recurrence plot set, its parameters need to be compared and verified. The learning rate, patch size, and optimizer are discussed as examples. ViT uses the parallelization method in the training process and the softmax cross entropy between the standard value and the model prediction value to calculate the average loss. This loss value is then reduced by using the Adam optimizer to decrease the error. To improve the model’s generalization ability, dropout regularization is added behind each sub-layer, and *P*_drop_ is set to 0.1.

(1) Learning rate

The learning rate directly influences convergence behavior, while inappropriate values may lead to slow training or unstable optimization. As shown in Table 4, the learning rate of 0.0001 has the highest learning rate accuracy compared to other values, and is therefore selected.

(2) Patch size

In ViT, the input image is divided into fixed-size patches for feature embedding. An excessively small patch size tends to increase model complexity and overfitting risk, whereas too large a size may weaken local information. According to Table 5, 32 × 32 patches have the best performance compared to other sizes, indicating an appropriate balance between local detail and global perception.

(3) Optimizer

The optimizer affects the efficiency of gradient learning. Comparative results in Table 6 show that Adam significantly outperforms stochastic gradient descent (SGD) due to its adaptive learning capability. Thus, Adam is adopted in the final configuration.

The optimal combination obtained from the above comparisons is summarized in Table 7 and used throughout subsequent experiments to ensure the robustness and accuracy of spatial feature extraction in the proposed method.

#### 5.2.3. Analysis of Fault Diagnosis Results

**Figure 19:**
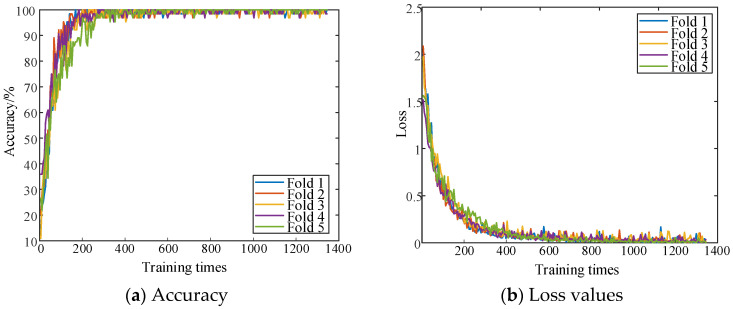
Accuracy and loss curve of the training set of the parallel two-channel model.

The parallel dual-channel model is trained by combining the characteristics of one-dimensional time-series signals and two-dimensional space images. Figure 19 shows the curve of the accuracy and loss value of the training set under the five-fold cross-validation method.

Figure 19 illustrates that as the number of training iterations increases, the loss value curve shows a trend of gradual decrease and stabilization. After approximately 600 iterations, the algorithm begins to converge.

**Figure 20:**
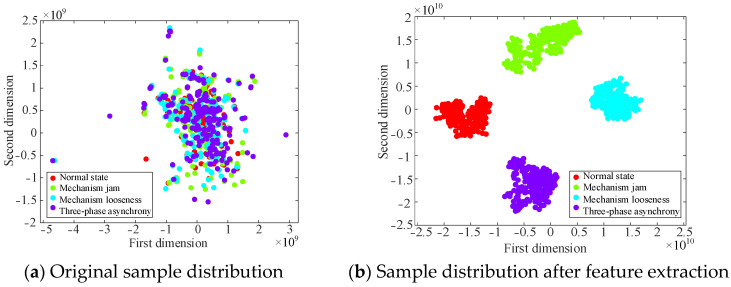
Visualization effect of t-SNE.

Then, t-distributed stochastic neighbor embedding (t-SNE) is introduced to provide a clearer visualization of the effects of the proposed method in extracting fault features. t-SNE is derived from SNE, addresses the issue of data point congestion in SNE, and is suitable for reducing high-dimensional data to two-dimensional (2D) or three-dimensional (3D) and for visual display. Compared with other dimensionality reduction methods, the advantage of t-SNE is that it can not only capture the local structure of high-dimensional data, but also retain some global structure. This study utilizes t-SNE to map high-dimensional data into a 2D space. The 2D plane can be distinctly represented without intricate interaction, which can effectively balance the retention of the local structure and the expansion of the global distribution. Although a 3D space has the capacity to store a greater amount of information, it may also lead to the inclusion of unnecessary details and increase the complexity of observation [30,31]. After introducing the t-SNE algorithm, the visualization results are shown in the fully connected layer, as shown in Figure 20.

**Figure 21:**
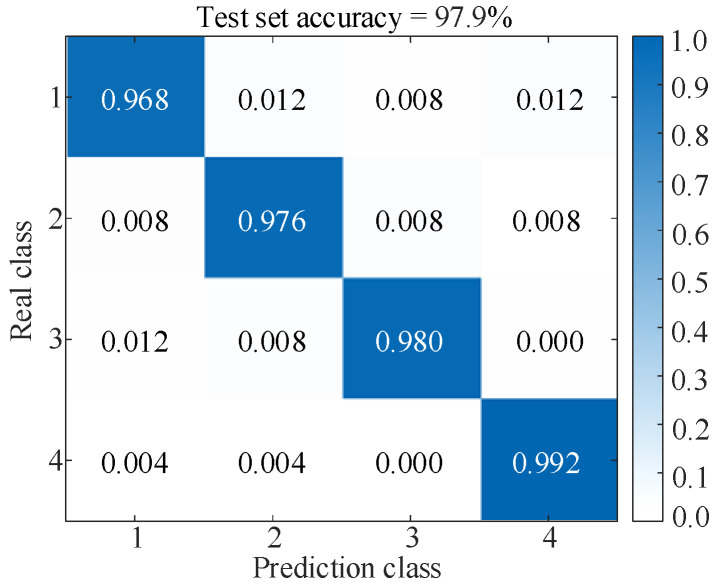
Results of the test set (Under the GW4-252 disconnector experiment).

Figure 20a shows that the original samples are scattered and have no obvious rules. After parallel dual-channel model feature extraction, Figure 20b illustrates a distinct border and linear separability, which reflects the superiority of the t-SNE algorithm, and shows that the model is capable of successfully differentiating characteristics across several categories, thereby improving the classification accuracy. The results of the test set after five-fold cross-validation are shown in Figure 21. Simultaneously, the values of precision, recall, and F1-score of the model evaluation index are obtained, as shown in Table 8.

Figure 21 and Table 8 show that the precisions for the four types of faults are 96.8%, 97.6%, 98.0%, and 99.2%, respectively, indicating that most of the predicted abnormal samples are actual anomalies with low false alarm rates. From the recall and F1 score under four types of faults, it can be seen that the model can successfully detect the vast majority of abnormal samples with extremely low false negative rates. Although the recognition performance of fault types 1~3 is slightly lower than that of fault type 4, all evaluation indicators remain above 96.00%, proving that the model still maintains a high degree of stability and robustness between different fault types. Overall, the results in Table 8 highlight the superiority of the proposed method in fault classification tasks, highlighting its feasibility and effectiveness when applied in complex and changing industrial environments.

#### 5.2.4. Comparison with Other Models

**Figure 22:**
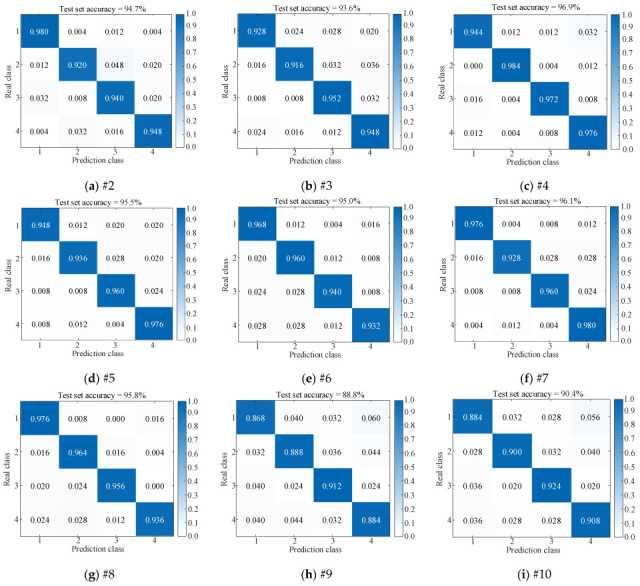
Comparison of confusion matrix results of several models.

**Figure 23:**
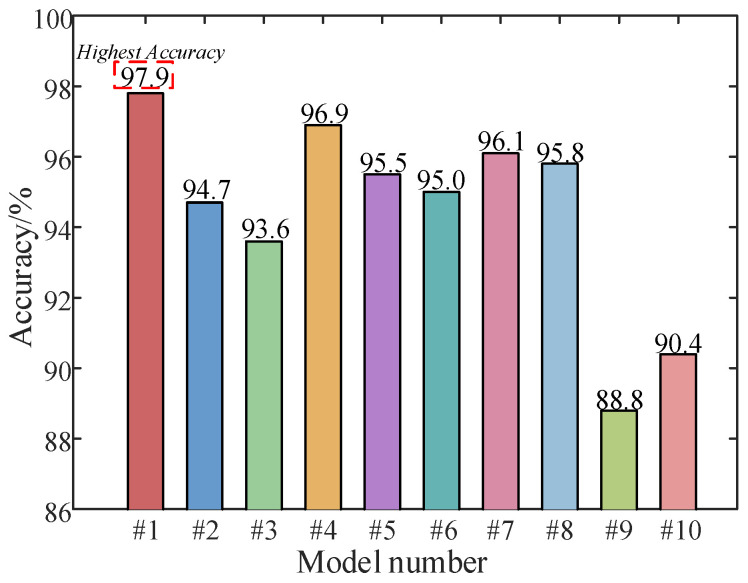
Comparison of model test results.

In this study, the proposed model is compared with several related models, and two classical fault diagnosis models are introduced: EMD—PE (Permutation Entropy)—SVM and VMD—SE (Sample Entropy)—ELM (Extreme Learning Machine). Each model is trained using five-fold cross-validation and tested using a test set to evaluate the effectiveness and accuracy of the proposed model. Table 9 presents the accuracy of the results. Figure 22 illustrates the results of the confusion matrix and Figure 23 illustrates a comparison of the test results.

The conclusions drawn from Table 9, Figure 22 and Figure 23 are as follows: From #1, #2, and #3, the parallel dual-channel model based on feature fusion can better extract fault features than the single-channel model, thus improving the fault recognition rate. Compared with #1 and #4, the image conversion of one-dimensional vibration signal by RP is better than that by GAF. Compared with #1 and #5, the effect of using ViT to extract spatial features is better than CNN, and the accuracy is higher. Compared with #1 and #6, GRU is better than LSTM in extracting time-series features. From #1, #7 and #8, the effect of using BKA to optimize VMD parameters is better than that of NGO and PSO, thus improving the recognition accuracy. From #1, #9 and #10, compared with the classical fault diagnosis algorithm, the fault recognition ability of the proposed model is much higher.

#### 5.2.5. Extended Verification of the Proposed Method

**Figure 24:**
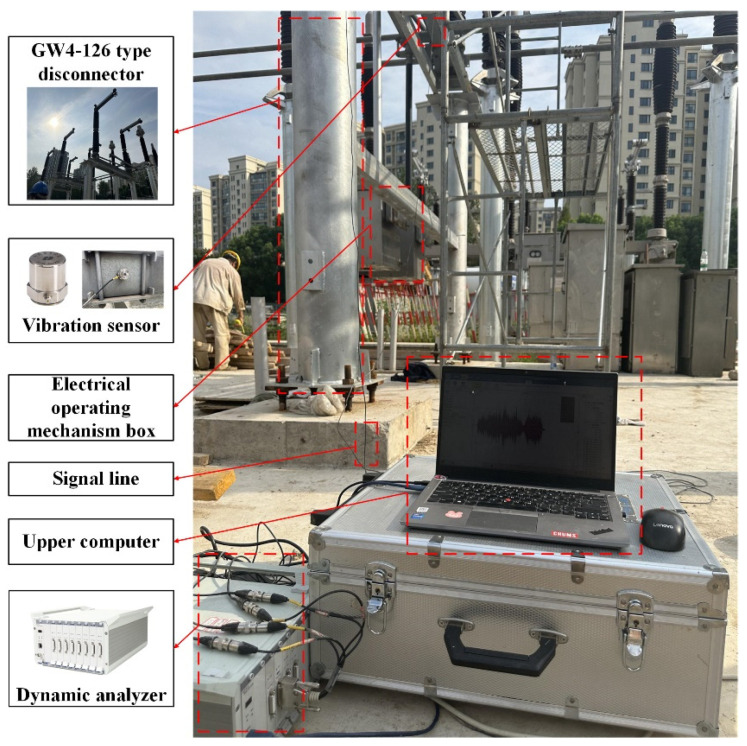
Vibration experimental platform of GW4-126 type disconnector.

The proposed model is extended and applied. Taking a GW4-126 disconnector (The equipment is produced by Shandong Taikai Disconnector Co., Ltd., and put into use in Nanjing, China) running in a 110 kV substation as an example, the experimental platform of the disconnector is built as shown in Figure 24.

**Figure 25:**
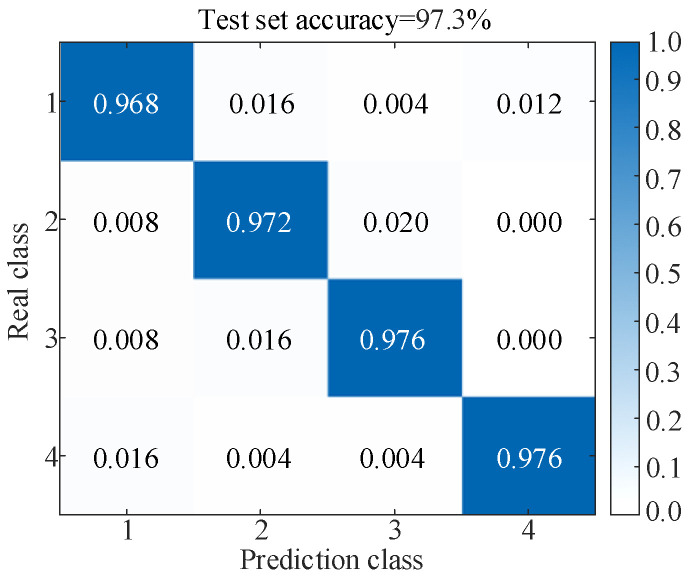
Results of the test set (Under the GW4-126 disconnector experiment).

The construction process of the experimental platform, the installation of the vibration sensor and the fault setting method, and the experimental process are the same as Section 5.1.1, Section 5.1.2 and Section 5.1.3. After collecting the vibration signals under four operating conditions, the results are analyzed, and the specific process is shown in Section 5.2. Finally, the fault diagnosis results are shown in Figure 25 and Table 10.

Figure 25 and Table 10 indicate that the proposed method is still effective for other types of disconnectors and has application value.

## 6. Conclusions

This study presents a parallel dual-channel method for diagnosing mechanical faults in disconnectors to enhance diagnostic accuracy. This approach is based on feature fusion, considering the characteristics of vibration signals associated with mechanical faults and leveraging the benefits of timing and spatial feature extraction models. The following conclusions can be drawn from the theoretical analysis and experimental verification.

1. The optimal value of parameter [*K*, *α*] of the VMD is obtained using BKA. Compared with GWO, GEO, CSA, PSO, SABO, DBO, NGO, GTO, and GA, the convergence effect is clearly better, and the uncertainty of the subjectively defined parameter values is avoided. By using the kurtosis value to screen out IMFs with richer fault information and reconstruct the signal, the transient impact of the fault signal can be precisely captured, thereby offering a more dependable foundation for fault diagnosis. The application of the GRU to extract time-series features mitigates the issue of gradient disappearance in RNN. Compared with LSTM, GRU reduces the information transmission path, improves the sensitivity to failure features, reduces the calculation complexity, and enhances the model’s generalization capability.
2. RP uses the correlation characteristics of time-series data to obtain feature information from its internal structure. Compared with GAF and other image coding algorithms, RP has higher accuracy. Combined with the higher-quality adaptive feature extraction capability of ViT for images, a fault diagnosis of the disconnector is conducted. This performance is superior to that of the traditional CNN.
3. By combining temporal and spatial features, the information of different modes can be comprehensively utilized to describe the characteristics of data more comprehensively, which improves the issue of inadequate recognition accuracy resulting from diagnosis only through a single data dimension, and then enhances the model’s stability and reliability. An ablation experiment is conducted in this study, and the proposed model achieves an accuracy of 97.9%, demonstrating superior robustness compared to other related methods.
4. Three methods are proposed for setting the mechanical fault of the disconnector and the optimal arrangement of the vibration sensor. Simultaneously, the vibration data of the disconnector is gathered under different operating conditions, and the mechanical fault of the disconnector is diagnosed. The issues of inadequate sample size for mechanical fault data of disconnectors and difficult simulation of mechanical faults of disconnectors are solved to some degree.

## Figures and Tables

**Table 1:** Comparison of existing mechanical fault diagnosis methods.

Method Category	Representative Techniques	Strengths	Limitations
Signal processing + classical classifiers	EMD, VMD + SVM, KNN	Effective for obvious faults; VMD: Addressing the issue of modal aliasing in EMD	Manual feature extraction required; Poor performance for similar early faults
Deep learning method on 2D images: signal-to-image conversion	RP, GAF, MTF	RP: Handles nonlinear signals; Works with small samples	GAF, MTF: Lack of ability to process vibration signals
Deep learning method on 2D images: spatial feature extraction	CNN, ViT	ViT: Strong global feature extraction; Efficient parallel computation	CNN struggles to process global information
Deep learning on 1D sequences	CNN, RNN, LSTM, GRU	GRU: Captures temporal dependencies; Simpler structure	CNN lacks temporal modeling; RNN trains slowly; LSTM structure is complex

**Table 2:** Initial parameters of BKA-VMD.

Population Size	Maximum Iteration Count	Value Range of *K*	Value Range of *α*
30	100	(1, 100)	(10, 3000)

**Table 3:** Parameter optimization results of for BKA-VMD.

Operating Conditions	*K*	*α*
Normal state	4	365
Mechanism jam	6	238
Mechanism looseness	3	1224
Three-phase asynchrony	5	1516

**Table 4:** Impact of different learning rates on the model.

Learning Rate	Accuracy/%
0.01	87.1
0.001	92.3
0.0001	98.0

**Table 5:** Impact of different patch sizes on the model.

Patch Size	Accuracy/%
8 × 8	93.8
16 × 16	96.4
32 × 32	97.2

**Table 6:** Impact of different optimizers on the model.

Optimizer	Accuracy/%
SGD	91.7
Adam	98.1

**Table 7:** Details of model parameters.

Parameter	Value
Learning rate	0.0001
Patch size	32 × 32
Optimizer	Adam
Number of attention heads	12
Image size	227 × 227
Multilayer perceptron size	3072

**Table 8:** Model evaluation index values (Under the GW4-252 disconnector experiment).

Tag Type	Precision/%	Recall/%	F1-Score
1 (Normal state)	96.8	97.6	97.2
2 (Mechanism jam)	97.6	97.6	97.6
3 (Mechanism looseness)	98.0	98.4	98.2
4 (Three-phase asynchrony)	99.2	98.0	98.6

**Table 9:** Comparison of the results of several models.

No.	Model Types	Accuracy/%
#1	RP-ViT+BKA-VMD-GRU	97.9
#2	RP-ViT	94.7
#3	BKA-VMD-GRU	93.6
#4	GAF-ViT+BKA-VMD-GRU	96.9
#5	RP-CNN+BKA-VMD-GRU	95.5
#6	RP-ViT+BKA-VMD-LSTM	95.0
#7	RP-ViT+NGO-VMD-GRU	96.1
#8	RP-ViT+PSO-VMD-GRU	95.8
#9	EMD-PE-SVM	88.8
#10	VMD-SE-ELM	90.4

**Table 10:** Model evaluation index values (Under the GW4-126 disconnector experiment).

Tag Type	Precision/%	Recall/%	F1-Score
1 (Normal state)	96.8	96.8	96.8
2 (Mechanism jam)	97.2	96.4	96.8
3 (Mechanism looseness)	97.6	97.2	97.4
4 (Three-phase asynchrony)	97.6	98.8	98.2

## Data Availability

The data that support the findings of this study are available from the corresponding author upon reasonable request.

## Abbreviations

VMD	Variational Mode Decomposition
BKA	Black-winged Kite Algorithm
IMF	Intrinsic Mode Function
GRU	Gated Recurrent Unit
RP	Recurrence Plot
ViT	Vision Transformer
EMD	Empirical Mode Decomposition
KNN	K-Nearest Neighbor
SVM	Support Vector Machine
GAF	Gramian Angular Field
MTF	Markov Transition Field
CNN	Convolutional Neural Network
RNN	Recurrent Neural Network
LSTM	Long Short-Term Memory
ADMM	Alternating Direction Multiplier Method
GWO	Grey Wolf Optimizer
GEO	Golden Eagle Optimizer
CSA	Crow Search Algorithm
PSO	Particle Swarm Optimization
SABO	Subtraction-Average-Based Optimizer
DBO	Dung Beetle Optimizer
NGO	Northern Goshawk Optimization
GTO	Gorilla Troops Optimizer
GA	Genetic Algorithm
2D	Two-Dimensional
3D	Three-Dimensional
PE	Permutation Entropy
SE	Sample Entropy
ELM	Extreme Learning Machine
